# Forests Regenerating after Clear-Cutting Function as Habitat for Bryophyte and Lichen Species of Conservation Concern

**DOI:** 10.1371/journal.pone.0018639

**Published:** 2011-04-07

**Authors:** Jörgen Rudolphi, Lena Gustafsson

**Affiliations:** Department of Ecology, Swedish University of Agricultural Sciences, Uppsala, Sweden; University Copenhagen, Denmark

## Abstract

The majority of managed forests in Fennoscandia are younger than 70 years old but yet little is known about their potential to host rare and threatened species. In this study, we examined red-listed bryophytes and lichens in 19 young stands originating from clear-cutting (30–70 years old) in the boreal region, finding 19 red-listed species (six bryophytes and 13 lichens). We used adjoining old stands, which most likely never had been clear-cut, as reference. The old stands contained significantly more species, but when taking the amount of biological legacies (i.e., remaining deciduous trees and dead wood) from the previous forest generation into account, bryophyte species number did not differ between old and young stands, and lichen number was even higher in young stands. No dispersal effect could be detected from the old to the young stands. The amount of wetlands in the surroundings was important for bryophytes, as was the area of old forest for both lichens and bryophytes. A cardinal position of young stands to the north of old stands was beneficial to red-listed bryophytes as well as lichens. We conclude that young forest plantations may function as habitat for red-listed species, but that this depends on presence of structures from the previous forest generation, and also on qualities in the surrounding landscape. Nevertheless, at repeated clear-cuttings, a successive decrease in species populations in young production stands is likely, due to increased fragmentation and reduced substrate amounts. Retention of dead wood and deciduous trees might be efficient conservation measures. Although priority needs to be given to preservation of remnant old-growth forests, we argue that young forests rich in biological legacies and located in landscapes with high amounts of old forests may have a conservation value.

## Introduction

A forest in a natural landscape is formed by disturbance events [1], in the boreal forest mainly in the form of fire [2], pest out-breaks (e.g. [3]), and wind-storms [4], [5]. In present-day production forests in Scandinavia, naturally occurring disturbances are unusual, primarily due to successful fire-prevention [6]. Artificial disturbances are, however, very frequent since a large part of the forest landscape is being managed, predominantly with the clear-cutting system, since the 1950s [7]. Biological legacies (e.g. dead and live trees) remaining after disturbance, are important drivers of biodiversity in regenerating forests [8]. Post-harvest forest succession differs significantly from succession after a natural disturbance because little or no above-ground legacy remains after clear-cutting, in contrast to most natural disturbances [9]. Many threatened forest species depend on logs, snags and old live trees [10], and a reduction in the amount of such substrates implies reduced survival possibilities for a number of species. Landscape properties are also key factors for the recovery of forest species after disturbance, for example surrounding old forests act as dispersal sources for species, and occurrence of wetland, may be essential for species that require high humidity.

Modern silviculture has caused the forest landscape of North Europe to change from being dominated by uneven-aged and heterogeneous stands to even-aged and homogenous stands [11]. In addition, the amount of old-growth forest and dead wood has decreased [12], causing the loss of important substrates for many forest-living species [10], [13]. Most forest biodiversity studies focus on old or mature forests and knowledge about the biodiversity in young forests is sorely lacking (but see [14]–[16]), although their potential ecological value is likely to be high [17]. In boreal Sweden, as in many parts of the circum-boreal region, many of the remnant old forests are being harvested at present, which means that the possibility for dispersal from old to young stands will be lower in the future. In fact, during the period from 2000 to 2005 the area of forest younger than 60 years increased by 217 000 ha per year to comprise ca 57% of the whole forest area in the Nordic/Baltic countries (excl. Iceland), whereas the area covered by forests older than 60 years decreased by 162 000 ha per year to comprise ca 43% of the forest area [18].

Bryophytes (mosses and liverworts) and lichens are important components of biodiversity in boreal and temperate forests [19], and in being poikilohydric (i.e. lacking roots and absorbing water and nutrients through their surface) many species depend on high precipitation and humidity [20], [21]. This means that many of them are sensitive to forestry [22], [23], and consequently some bryophyte and lichen species in production forest landscapes are mainly confined to old-growth forest remnants [22]. Several studies have revealed a higher diversity and abundance of bryophytes and lichens in natural compared to managed forests [24]–[26]. The explanation to this pattern has been suggested to be either species' dispersal inefficiency [27], [28] or micro-climatic constraints [29]. Alternatively, differences in the amount of substrate available, with significantly more dead wood and old trees in the old-growth forests, may serve as a reason why the number of species is higher in natural forests [30], [31]. Transplantation experiments of old-growth forest lichens into young forests indicate that these species can survive there, suggesting that they are limited by their inability to either establish or disperse [32]. Not only have forestry operations been shown to affect bryophytes and lichens negatively in the managed stands, but also in the adjoining stands. Negative edge effects are, however, dependent on the cardinal location of clear-cuts; in the northern hemisphere with fewer species and a stronger decline in growth at south- than north-facing edges [33]–[35]. How edge orientation affects species in the young stands bordering to old stands is, nonetheless, largely unknown.

The main aim of this study was to investigate if young stands regenerating after clear-cutting can function as habitat for red-listed bryophytes and lichens. We hypothesized that the amount of available substrates (dead and living trees) remaining from the previous tree generation would be of great importance for the occurrence of the investigated species in the young stands. As a reference, we used old stands that probably never had been clear-cut, and by selecting pairs of young and old stands adjoining each other we could also investigate possible species dispersal from the old to the young. We also tested if area of old forest and wetland in the surroundings were important for the occurrence of red-listed bryophytes and lichens.

## Methods

### Stands

The forest stands were situated in the transition between the southern and middle boreal vegetation zones [36] on land owned by the forest company Holmen Skog, Sweden, comprising an area of approximately 4,700 km^2^, centred at 61° 57′ N, 16° 30′ E.

**Figure 1:**
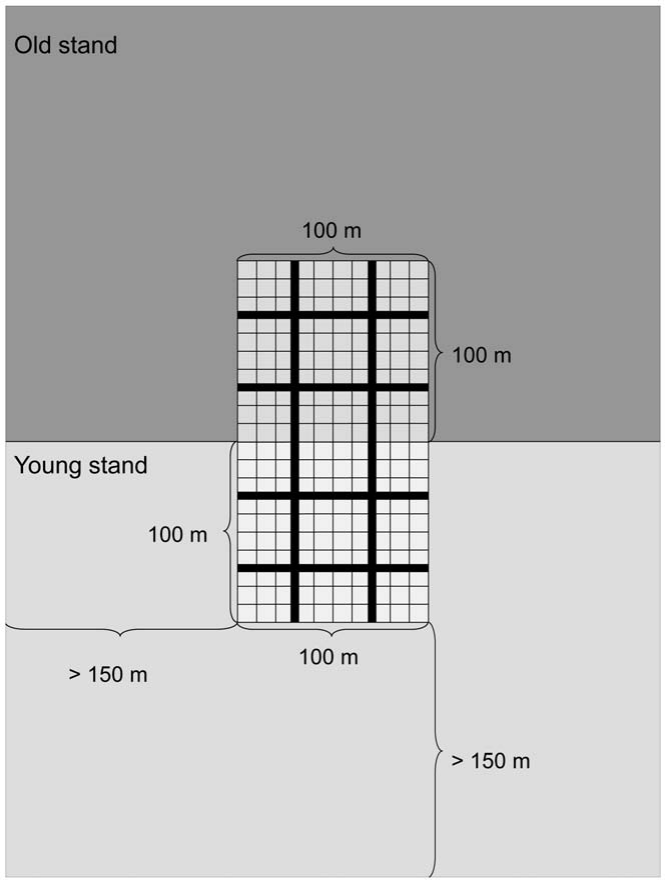
Schematic figure of the sampling design. Species were surveyed in subplots (small grid cells) and dead wood and deciduous trees in transects (black bands) in old (dark grey) and young (light grey) forest stands. The plots in the young forests were all more than 150 m from nearest other old forest.

All stands larger than 3 ha, less than 400 m above sea level, composed of at least 50% *Picea abies* (L.) Karsten by volume, and within either of the two age groups: 1) young stands 30–70 years old and 2) old stands >95 years old, were selected from the stand database of the landowner. In total 19 stand pairs, in which a 100×100 m plot could be positioned on both sides of the border between young and old stands were found in the area (Fig. 1). Stand pairs, in which the plot in the young stand could not be positioned further away than 150 meter from old stands other than the adjoining stand we aimed at surveying, were omitted as were stands with the exotic *Pinus contorta* Douglas ex Loudon. Several a priori delineated 100×100 m plots were not possible to follow exactly in the field, due to the presence of small streams, extraction roads, partial cuttings, etc. In such cases, minor adjustments were made in order to achieve a 100×100 m study plot, and since the borders between the young and old stands were not always straight, the total investigated area was 17.7 ha for old and young forests respectively, instead of 19 ha. The young stands were previously clear-cut and planted with *Picea abies*. These stands were now single-layered, and the trees within the stands were even-aged. Since clear-cutting was introduced in Sweden 60–70 years ago, the old stands had most likely never been clear-cut. As a consequence, the age of the old forests in the stand data-base is an underestimation; it does not correspond to the age of the stand, but instead to the age of the dominating tree layer. Tree cover continuity could be considerably longer, and thus true stand age considerably higher. Since all young stands had regenerated after clear-cutting, their age reflected true stand age.

### Species and substrate inventory

Each plot was divided into 10×10 m subplots, which formed the units of observation. We searched each subplot for presence of red-listed bryophytes and lichens [37] on all substrates (ground, trees, boulders etc) from the ground up to 2 m. The liverwort *Lophozia ciliata* Damsh. L. Söderstr. et Weib. (excluded from Table S1) was found in so many subplots that it, due to time constraints, had to be omitted from the survey.

For the same reason also the lichen *Micarea globulosella* (Nyl.) Coppins was not possible to record in detail, and thus only its presence per plot was noted. The survey of all re-listed bryophytes and lichens would have suffered from the disproportionately large effort needed to record these two species in the same detail. All red-listed bryophytes and lichens in the study region were in the Red List described as being negatively affected by logging, and/or by a decrease in substrates associated with forestry activities [38], [39]. Since dead wood and deciduous trees are key components for biodiversity in boreal forests, we performed detailed surveys of these structures in the plots. We used the Line Intersect Sampling method to estimate amount of dead wood [40] with four line transects placed in each plot (Fig. 1). Three classes of degree of decay were used: 1) hard wood (0–10% of the trunk volume consists of soft, decayed wood); 2) moderately decayed wood (11–75% decayed wood; still with a heart of hard wood); 3) well-decayed wood (76–100% decayed wood; a pointy object is possible to push through the entire trunk). The volume and surface area of downed wood per plot were calculated according to Gregorie & Valentine [41]. Standing dead wood (snags and stumps) were measured within two metres on either side of each transect, as was the diameter at breast height of each *Populus tremula* L., *Sorbus aucuparia* L. and *Salix caprea* L. tree standing. Since other deciduous trees like *Betula pendula Roth., Betula pubescens* Ehrh., and *Alnus incana* (L.) Moench only rarely act as hosts for the species surveyed in this study, these were not investigated in detail. The size of the deciduous trees and the degree of decomposition of dead wood in the young stands revealed that most of such substrates were legacies from the previous forest generation.

### Surrounding landscape

To analyze the impact of forest age and prevalence of wet areas in the surrounding landscape on red-listed species in the young stands, three virtual buffer zones were created using a geographic information system (ArcGis 9.1). The buffer zones were centred in the young plots and reached 100, 200 and 400 meters, respectively, from the plot edge. Within these buffer zones the area of old forests was measured using kNN (k- Nearest Neighbour) satellite mapping, with a 25×25 m resolution. To the area present old stands we added the area of forests younger than our focal young stands. The rationale for this was that forests that were younger than our inventoried young stands were old at the time of harvest of our focal stands, and may thus have acted as propagule sources before being clear-cut. The remote sensing kNN-data is calibrated against field data from the National Forest Inventory, i.e. the annual national field sample of forest variables [42].

The prevalence of wetlands, defined as areas of permanent soil water-saturation, within 100 and 400 metres, respectively, of the young stand plots was measured in ArcGis from the terrain map (1∶25 000). The direction of each young stand in relation to their adjacent old stand was recorded from maps in ArcGis 9.1. The young stands were then put into one of two cardinal position groups, N and S (where N-stands were located E, NE, N or NW and S-stands were located W, SW, S or SE of the old stand). Nine stands were in the north-facing and 10 in the south-facing group. When analyzing the effect of cardinal direction of the border, only the band 0 to 20 m in the young stands was used, since any shading effect of the old forest is likely to be insignificant further out in the stand. Altitude measurements were obtained from the forest company stand database.

### Statistical analysis

The number of red-listed species and observations (number of subplots in which the species were encountered), as well as the amount of dead wood and number of deciduous trees were compared pair-wise between the young and old stands using Wilcoxon signed rank tests. We used Chi-Square-test to analyze compositional differences between young and old stands in respect to proportions of different decay stages of dead wood. In order to take into account possible differences in substrate amounts between the young and the old stands, we used sample-based rarefaction curves [43] in the program EstimateS [44] for the comparison of species density of bryophytes and lichens. We rescaled the x-axes to represent the main substrates for the species studied: cumulative surface area of dead lying coniferous trunks for epixylic bryophytes (in this analysis only liverworts), and number of living deciduous tree stems with a diameter at breast height larger than 10 cm for epiphytic lichens. For the rescaling of the x-axis we standardized substrate area based on average study site values of surface area of dead lying coniferous trunks or number of living deciduous tree stems in the young and old stands separately. We then visually compared curves at comparable levels of sampling effort, i.e. at an equal cumulative log area or number of stems sampled. Differences in species richness were considered not significant (p>0.05) if confidence intervals according to Colwell et al. [45] overlapped. Only bryophytes confined to growing on dead wood and lichens exclusively found on deciduous trees were analyzed in this way.

When analyzing the edge effects of borders between young and old stands, we divided both adjacent 100×100 m plots into five 20 m-wide bands, parallel to the common edge. The bands positioned furthest from the border, i.e. 80–100 metres, were omitted because the shape of some stands did not accommodate sufficient sub-plots within that band. Analyses of species richness and number of observations were made for young and old stands using log-linear regression with a logarithmic link function [46]. Poisson distribution of errors was fitted and where the response variables showed signs of overdispersion (i.e. the variance being larger than the mean), we used a negative binomial distribution of residuals. The dispersion was checked for every run and the model having a quota between Pearson Chi-Square and the number of the degrees of freedom closest to unity was chosen. In addition, we used Generalized Estimating Equations (GEE) [47], which is less sensitive to the distribution imposed on the data [48], and recommended when analyzing data collected in clusters where observations within a cluster may be correlated [49]. First, we tested the hypothesis that a linear model could describe the pattern in number of species or observations in relation to the distance to the edge. If this was not the case, we tested if the reason for this was a break in the linear model at the border between the old and young stands.

The effects of six environmental variables on the number of species and observations of lichens and bryophytes in the young stands were tested using Poisson or negative binomial regression with logarithmic link function, as above. Five of these were landscape variables, for bryophytes: the area of old forests and wetlands in the surrounding landscape, altitude, latitude, species richness or number of observations in the adjoining old forest. The within-plot variable was surface area of coniferous logs ha^−1^, since this was the main substrate for a majority of the bryophyte species found. For lichens the same landscape variables were included, but no within-plot variable. All variables were included in the original model for bryophytes and lichens, respectively. The models were then simplified using stepwise variable selection to minimize the AIC (Akaike's information criterion), adjusted for small samples as recommended by Burnham & Anderson [50]. The three different buffer zones for old forests and two buffer zones for wetlands in the surrounding landscape were included separately in the initial models and the model with the lowest AIC_c_ was used as starting model. Since a Type 3 analysis was used, the order in which the terms for the model were specified had no effect.

For easier interpretation of the results, all estimate values were back-transformed from the original model estimates: E_t_ = Exp(E_m_), where E_m_ is the model estimate and E_t_ is the back-transformed value. This expresses the proportional change in the response variable per unit change in the predictor variable, given that all other predictor variables are held constant. For the statistical analyses, we used the software SAS 9.1.

## Results

### General stand characteristics

Mean stand age according to the stand data-base of the forest company was 44 years for the young and 105 years for the old. The mean proportions of *Picea abies* were 73% and 75%, of *Pinus sylvestris* L. 16% and 12%, and of deciduous trees 13% and 11%, in young and old stands, respectively. There were no significant differences in any of these proportions between the age classes. The habitat variation regarding soil and topography (site index, soil-moisture regime, ground structure) was similar between young and old stands (Table 1).

**Table 1:** Species occurrence and stand characteristics.

	All stands; n = 19			North-facing edges; n = 9^a^			South-facing edges; n = 10^a^		
	young	old	p-value	young	old	p-value	young	old	p-value
No. of bryophyte species	1 (0–3)	2 (0–4)	**0.001**	0 (0–1)	1 (0–2)	0.203	0 (0–1)	1 (0–2)	**0.008**
No. of bryophyte observations^b^	1 (0–7)	6 (0–38)	**0.001**	0 (0–1)	1 (0–4)	0.125	0 (0–1)	2 (0–8)	**0.008**
No. of lichen species	2 (1–6)	5 (2–7)	**0.001**	1 (0–2)	1 (0–2)	0.809	1 (0–2)	2 (1–3)	**0.014**
No. of lichen observationsb,c	2 (0–10)	4 (0–31)	**0.007**	0 (0–2)	1 (0–3)	0.273	0 (0–1)	1 (0–6)	**0.006**
No. of observations of *B.nadvornikiana* b	11 (0–64)	7 (0–47)	**0.031**	0 (0–11)	2 (0–9)	0.844	2 (0–5)	1 (0–8)	0.361
Surface area deciduous trees ha^−1^	0 (0–278)	27 (0–1079)	**0.007**	0 (0–278)	27 (0–427)	0.156	0 (0–48)	48 (0–1079)	0.555
Surface area coniferous logs ha^−1^	41 (0–418)	334 (44–968)	**0.002**	49 (0–402)	238 (44–968)	**0.004**	0 (0–418)	505 (206–891)	**0.002**
Surface area deciduous logs ha^−1^	0 (0–285.1)	118.2 (0–664.7)	**<0.001**	4 (0–285)	89 (0–354)	**0.016**	0 (0–91)	133 (0–665)	0.062
Prop. *Picea abies* (%)^e^	80 (51–91)	80 (55–100)	0.422	80 (51–91)	80 (55–100)	0.844	71 (53–80)	75 (60–89)	0.359
Prop. *Pinus sylvestris* (%)^e^	10 (0–40)	10 (0–30)	0.888	10 (0–40)	10 (0–30)	0.812	10 (0–35)	13 (0–30)	0.789
Prop. deciduous trees (%)^e^	10 (0–40)	10 (0–40)	0.531	9 (0–39)	10 (0–30)	0.625	16 (0–40)	6 (0–40)	0.357
Site index:number of stands d,e	G20:3; G21:4 G22:3; G23:3 G24:3; G26:3	G20:3; G21:1 G22:4; G23:5 G24:2; G25:1 G26:2; T22:1	0.802	-----	-----	-----	-----	-----	-----
Ground moisture class:number of stands^e^	Mesic:16; Moist: 3	Mesic:16; Moist: 3	-----	-----	-----	-----	-----	-----	-----
Ground structure class:number of stands^e^	Even:16; Somewhat uneven:3	Very even:1; Even:16; Somewhat uneven:2							

Pair-wise tests for differences between young and old forest stands using Wilcoxon signed rank test. Values represent median and range (within parentheses).

a Number of species or observations in the young stand 0-20m from the border to the old stand, and average number of species/observations per band in the old stand.

b One observation  =  presence in a 10×10 m plot.

c Excluding *Bryoria nadvornikiana* and *Micarea globulosella*. *M. globulosella* was too common to be recorded in detail.

d Differences in site index was tested using Fischer's exact test (excluding T22).

e Data from the forestry company data base.

### Dead wood and deciduous trees

Both the volume and surface area of dead wood differed significantly between young and old stands, with an average total coniferous log volume of 21 m^3^ ha^−1^ in the old stands and four m^3^ ha^−1^ in the young (Wilcoxon signed rank test, p<0.001), and a surface area of 118.6 m^2^ ha^−1^ in the old stands and 17.2 m^2^ ha^−1^ in the young (Wilcoxon signed rank test, p = 0.002). No significant difference in frequency of the different decay stages could be detected (Chi-2 = 3.56, p = 0.17, df = 2). The mean number of deciduous trees (excluding *Betula* spp and *Alnus incana*) in the old stands was 21 and in the young 4.4 (Wilcoxon signed rank test, p = 0.042).

### Number of species and observations

#### Bryophytes

In total, eight red-listed bryophyte species (six liverworts and two mosses) were found, representing 32% of the known red-listed forest bryophytes in the county of Gävleborg (Table S1). Six species were found in the young and all eight in the old plots.

**Figure 2:**
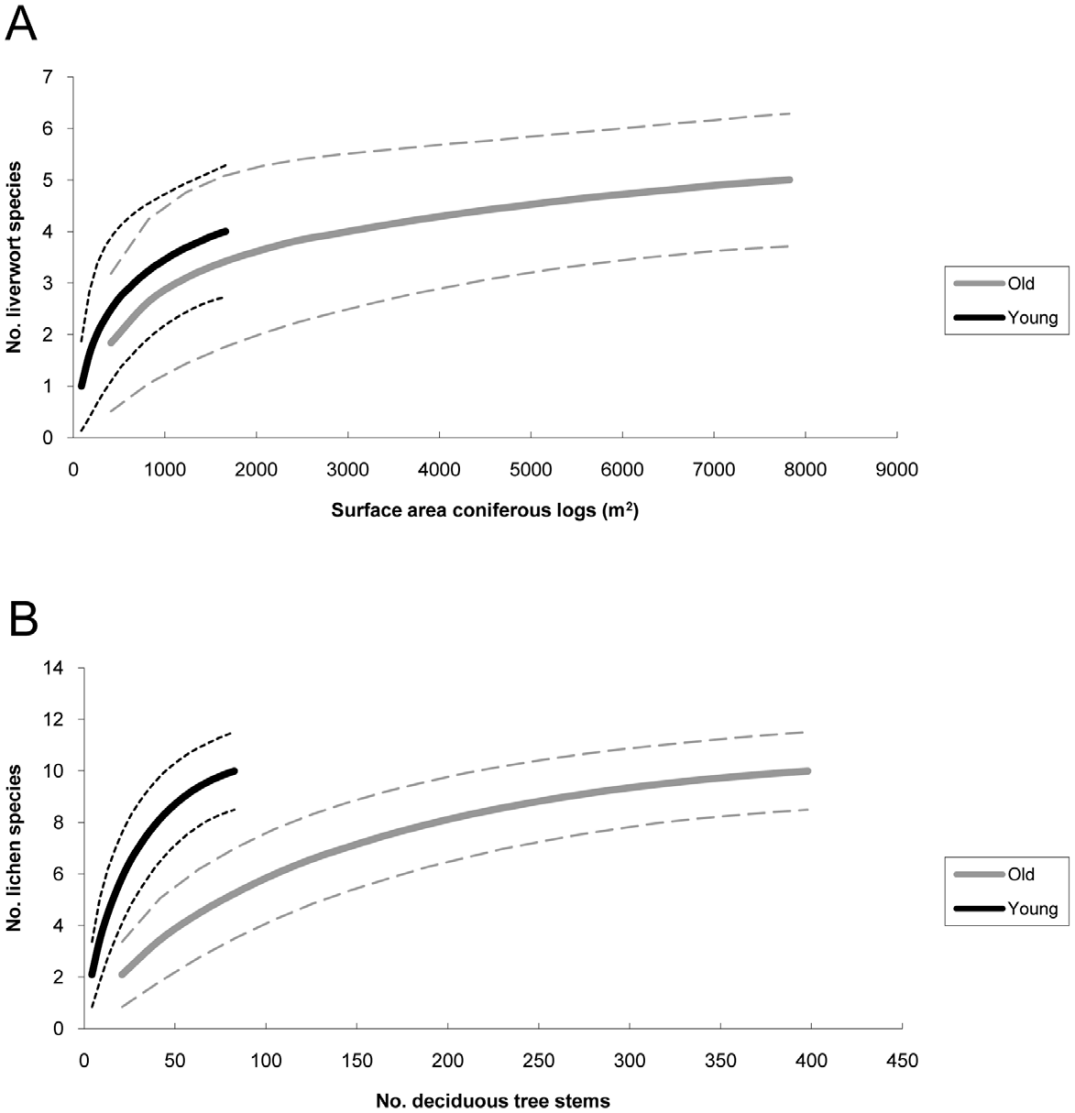
Sample-based rarefaction curves. Species density of red-listed species between old and young production forests of a) epixylic liverworts per surface area coniferous logs, and b) epiphytic lichens per deciduous tree stem. Confidence limits calculated according to Colwell et al. [45] are drawn as dashed lines.

The number of red-listed bryophyte species per plot varied between zero and four for old stands and zero and three for young stands. The number of observations per stand varied between zero and 38 in the old plots and zero and seven in the young. Mean species richness and number of observations were significantly higher in the old plots (both p<0.001) (Table 1). After standardizing by dividing the number of observations per area coniferous log, no differences could be detected for red-listed species associated with this substrate (Wilcoxon signed rank test, *p* = 0.177). Nor could any difference in species richness be detected when comparing equal surface area of logs, using rarefaction analysis (Fig. 2a).

#### Lichens

In total, 17 red-listed lichen species were found, comprising 21% of the known red-listed forest-living lichens in the county of Gävleborg. Thirteen species were found in the young and 16 in the old plots. The richness of red-listed lichen species varied between two and seven for old plots and one and six for young, and the mean species richness per hectare was significantly higher in the old (Table 1). The mean number of observations was 19 in the old and 23 in the young stands, and this difference was not significant. When excluding *Bryoria nadvornikiana* (Gyeln.) Brodo et. D.Hawksw. that was significantly more common in the young than the old stands, there was significantly higher number of lichen species observations in the old than in the young stands (Table 1). After standardizing for the total surface area of deciduous trees, no significant differences could be detected for the number of observations of red-listed lichens associated with this substrate (Wilcoxon signed rank test, *p* = 0.102). On the contrary, species richness was significantly higher in the young plots when equal numbers of deciduous trees were compared, as revealed in rarefaction analysis (Fig. 2b).

### Species' substrates

Almost all liverwort observations (91%) were made on lying, dead coniferous trees, mainly *Picea abies* (66%). The two moss species were found on *Populus tremula* and *Salix caprea*. All lichens, except one *B. nadvornikiana* individual, were found on either living or dead trees. With the most common species, *B. nadvornikiana*, excluded 64% of all observations were made on living trees, with the rest being on dead trees, and of the observations made on living trees, 94% occurred on deciduous trees.

### Effects of landscape variables

Bryophyte species richness was explained by the area of old forests within 200 metres. Conversely, lichen species richness was not significantly associated with any environmental factor. Number of bryophyte observations was explained by the area of both old forests and wetlands within 100 metres (9 ha) and also by number of bryophyte observations in the adjoining old plot (Table 2). Number of lichen observations was explained by the area of old forests within 100 metres.

**Table 2:** Score statistics from log-linear regression.

	No. of bryophyte species		No. of bryophyte observations		No. of lichen species		No. of lichen observations (excl. *B. nadvornikiana*)		No. of observations of *B. nadvornikiana*	
	estimate^a^	p-value	estimate^a^	p-value	estimate^a^	p-value	estimate^a^	p-value	estimate^a^	p-value
Area old forests 100 m			1.46	**0.009**			1.18	**0.029**		
Area old forests 200 m	1.16	**0.009**								
Area old forests 400 m					1.04	0.188				
Area wetlands 100 m			2.80	**0.029**						
Area wetlands 400 m										
Number of species/observations in corresponding old stand^b^			0.94	**0.011**					1.09	**0.035**
Altitude					0.99	0.178				
Latitude	1.00	**0.026**	1.00	**0.042**	1.00	0.097	1.00	**0.038**	1.00	0.081
Surface area coniferous logs m^3^ha^−1^			1.00	0.052	not tested		not tested		not tested	

Estimates and p-values are shown for the variables included in the final models. Also non-significant variables were sometimes included as a result of the model simplification using AIC_c_.

a Back-transformed from model values. The back-transformed values indicate the proportional change in the response variable per unit change in the predictor; i.e. an estimate of 1.5 responds to an increase in 50% in species number per unit increase in the predictor variable. A value on the estimate of 0.5 corresponds to a reduction in richness with half of the species present.

b Species number in the adjoining old stand when analyzing species richness and number of observations when analyzing number of observations.

**Figure 3:**
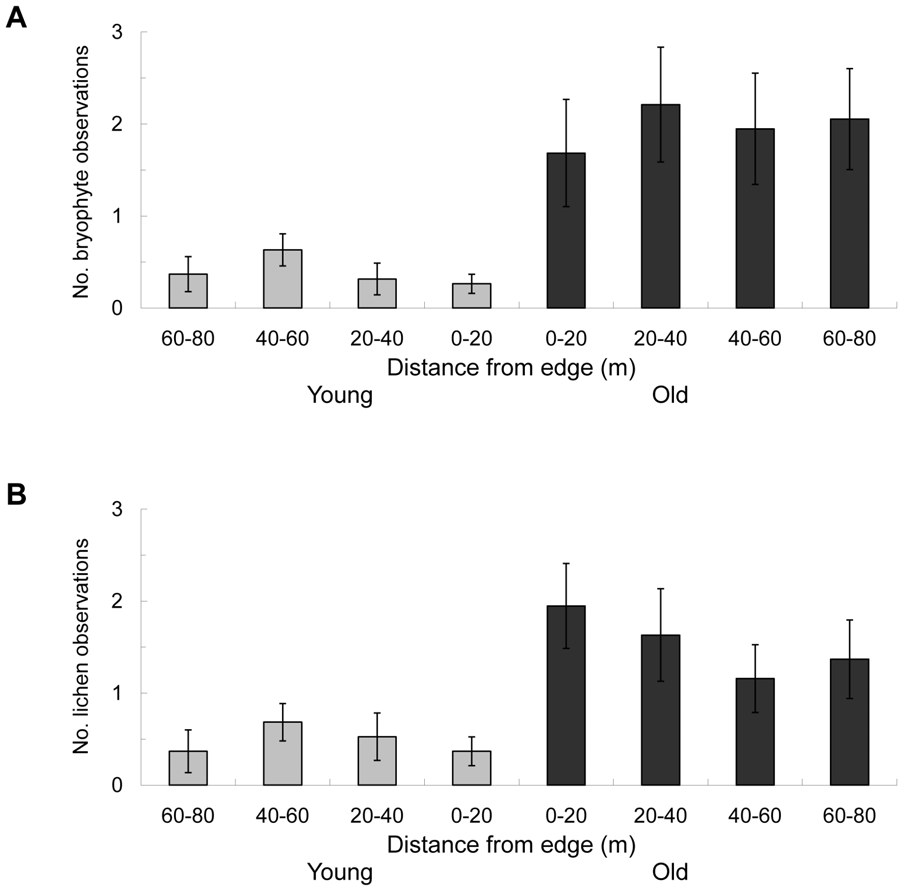
Edge effects. Number of subplots (10×10 m) where observations of red-listed bryophytes (A) and lichens (B) were recorded at different distances from the edge. Error bars indicate standard error.

Latitude, which was included in the final regression models for all response variables, significantly explained bryophyte species richness, as well as number of both bryophyte and lichen observations. This was due to the fact that the three southernmost stand pairs were outliers considering latitude, low species richness and low number of observations. When those stands were deleted, latitude was no longer significant. The remaining environmental factors showed a similar effect, regardless if the three outliers were included in the analysis or not. No significant effect of the distance to the edge could be detected in either the young or the old stands, using a log-linear regression approach (Fig. 3). When analyzing the impact of cardinal position, there were significant differences, with higher number of bryophyte and lichen species as well as observations in the old stands, when young stands were positioned south, but not north, of old stands (Table 1).

## Discussion

A main result from this study was that red-listed bryophytes and lichens may occur in young boreal production forests regenerating after clear-cutting. Nineteen such species were found, representing ca. 18% of all forest species of these organism groups that were on the Red List at the time of inventory, although total surveyed area was only 17.7 ha. Compared with old forests, the number of species and observations was generally lower, and this was not unexpected since the aim of the Red List is to identify species that are rare or declining, and such species are in production forest landscapes mainly confined to old forests with natural characteristics.

More surprisingly, species richness of epiphytic lichens was higher in the young stands when the same amount of deciduous host trees was compared, and there was no significant difference in bryophyte richness when amount of lying dead coniferous trees was compensated for. Nor was there any significant difference in the number of species observations, for either red-listed bryophytes or lichens, at equal substrate amounts. This points to another key result from the study; biological legacies from the previous forest generation are essential habitats for the red-listed bryophytes and lichens. The importance of available substrates has also been shown to be crucial for the lichen and bryophyte communities in naturally afforested, former arable land in comparison to permanent forestland in Estonia [51], and in logged stands in comparison to un-logged stands in British Colombia, Canada [52], but then mostly for common species. Maintaining host tree species diversity and retaining large or old stems of hardwoods have also been suggested as a means for sustaining epiphytic bryophyte diversity when managing forests in north-eastern U.S.A. [53].

Although many red-listed species found did occur on remnant live and dead trees, some species were also observed on young spruce trees that evidently represents new substrates. This is in agreement with the few studies that have investigated the occurrence of bryophytes and lichens, presumably associated with late-successional or old-growth forests, outside of old forests [54], [55]. These studies indicate that the environment in thinned or logged forest in fact is not unsuitable for such species. One example of a species on young spruce trees is the red-listed lichen *Bryoria nadvornikiana*, which had significantly more observations in the young than the old stands. This species obviously has high colonization ability in young Norway spruce production forests, and its qualification as red-listed consequently may need re-consideration.

In the ideal case, epiphyte inventories should embrace whole trees, from the stem base to the crown. But, since such methodology is difficult, laborious, and expensive, it is rarely applied (but see [56]). Instead it is common to restrict recordings to the lower two meters, as we did in our study. High irradiation promotes the growth of forest lichens, as long as the water availability is high enough for their metabolic activity [57]. Consequently, the light environment close to the ground in young, rather open forest is likely to be more beneficial to many species than that of old, dark and closed forest. Thus, it could be that many lichens in old stands are found higher up in the canopy and accordingly that their occurrence was underestimated in our study.

The ability of species to colonize and establish in young stands after logging is crucial for the species composition in future production forest landscapes. Dispersal capacity of crustose lichens is poorly known, and that of macrolichens has been shown to be highly variable [58]–[60], making results from studies of multiple species difficult to interpret. For colonizations on young spruce trees, which was observed for e.g. *B. nadvornikiana* and *Micarea globulosella*, dispersal from the surroundings is the only likely explanation, since old spruce trees with possible remnant populations were lacking. Spatial aggregation of the species was indicated by the positive correlations of number of bryophyte and lichen observations in the young stands to area of old forest within 100 m but not within the plot, i.e. less than 100 m. The study design used in this study does not allow for further interpretations on potential dispersal limitations within the spatiotemporal interval of 35–70 years and 100 m. However, Hylander [14] found no increase in colonization close to propagule sources on a scale of less than 100 m. The fact that we found a spatial aggregation of species observations when including the stands that might have acted as dispersal sources during the entire time period from the young stands were clear-cut, stresses the importance of the historical stand structure, also shown by Snäll *et al.* [61]. Longer studies that follow the development after clear-cutting are necessary to obtain a more thorough understanding of survival and dispersal patterns.

The lack of difference in both bryophytes and lichens species-richness and frequency of occurrence in young stands located to the north of old stands, was most likely due to the comparatively higher shade and humidity in this position. For bryophytes this was also indicated by the correlation of wetlands in the surroundings. At least for bryophytes, however, the differences between young and old stands bordering each other seem to level out, also at south-facing edges, when the young forest reaches ages of up to 50 years [14].

### Conclusion

Habitat degradation and destruction along with fragmentation of remaining habitat are major threats to biodiversity [62], [63]. Our study highlights the need to look beyond old conceptions about what constitutes the habitat of a species. We show that there is a potential for sensitive species to occur in young production forests, but that this largely depends on the retention at logging of structures like dead wood and deciduous trees, and also on the history of the forest landscape. Most of the boreal Nordic coniferous forests that are mature for harvest have previously been only selectively cut, first through high-grading and later by repeated thinnings. These forests still have traces of natural characteristics and many have tree-layer continuity. The colonization possibilities for their associated flora and fauna were very different compared with the situation in today's more fragmented landscape. The young forest stands in our study belong to the first generation after clear-cutting and are shaped by large-scale, highly mechanised forestry operations. Because modern forestry was introduced earlier in Sweden than in most other boreal countries, these young stands might indicate what will happen in other parts of the world, where forests have begun to be clear-cut more recently. Furthermore, should the clear-cutting system continue in future generations, there is a risk that the sensitive species will continue to decline. It is also likely that this decline will occur more rapidly than at present, since every clear-cutting is likely to decrease the amount of critical substrates.

Our study also indicates that biological legacies like dead and deciduous trees are important for sensitive bryophytes and lichens, and thus, that retention of such structures can be an efficient conservation tool. Since shade and high humidity often is beneficial to sensitive species, the location of retained trees is also important to consider. Additionally, in forest landscapes where future rotations are planned following clear-cutting, it might be necessary to increase the retention levels considerably, in order to counteract the expected successive decrease in species populations. Finally, we advise that young plantation forests in landscapes with large amounts of remnant natural forest characteristics could be incorporated into reserve networks, especially if they are located close to, and preferably north of old forests.

## Supporting Information

Table S1 List of bryophyte (liverworts and mosses) and lichen species registered in the old (n = 19) and young stands (n = 19).
(DOCX)
