# Supplementary material for: Forests Regenerating after Clear-Cutting Function as Habitat for Bryophyte and Lichen Species of Conservation Concern
Source: PLoS One. 2011 Apr 7;6(4):e18639. doi: 10.1371/journal.pone.0018639 (PMC3072405; doi:10.1371/journal.pone.0018639)
Supplement: Table S1 — List of bryophyte (liverworts and mosses) and lichen species registered in the old (n = 19) and young stands (n = 19). (DOCX) [file pone.0018639.s001.docx]

Table S1. *List of bryophyte (liverworts and mosses) and lichen species registered in the old (n=19) and young stands (n=19).*

|  | Species | Red-list category ^a^ | Old stands | | Young stands | |
| --- | --- | --- | --- | --- | --- | --- |
|  |  |  | No. of observations^b^ | No. of stands^c^ | No. of observations^b^ | No. of stands^c^ |
| Mosses | *Neckera pennata* | NT | 1 | 1 | - | - |
|  | *Orthotricum gymnostomum* | NT | 23 | 7 | 3 | 1 |
| Liverworts | *Anastrophyllum hellerianum* | NT | 43 | 9 | 10 | 5 |
|  | *Calypogeia suecica* | VU | 4 | 2 | - | - |
|  | *Cephalozia catenulata* | NT | 1 | 1 | 1 | 1 |
|  | *Lophozia ascendens* | NT | 16 | 7 | 3 | 2 |
|  | *Lophozia longiflora* | NT | 100 | 16 | 21 | 11 |
| Lichens | *Arthonia incarnata* | EN | 5 | 3 | 1 | 1 |
|  | *Biatora ocelliformis* | DD | 49 | 14 | 16 | 7 |
|  | *Bryoria nadvornikiana* | NT | 235 | 17 | 395 | 16 |
|  | *Calicium adaequatum* | NT | 4 | 2 | 4 | 1 |
|  | *Chaenotheca gracillima* | NT | 22 | 7 | 9 | 4 |
|  | *Chaenotheca laevigata* | VU | 4 | 3 | 2 | 1 |
|  | *Chaenothecopsis viridialba* | NT | 2 | 2 | - | - |
|  | *Cheiromycina flabelliformis* | VU | 10 | 4 | - | - |
|  | *Cladonia parasitica* | NT | 7 | 4 | 6 | 3 |
|  | *Cliostomum leprosum* | VU | - | - | 1 | 1 |
|  | *Collema furfuraceum* | NT | 2 | 1 | 1 | 1 |
|  | *Collema occultatum* | NT | 5 | 4 | 1 | 1 |
|  | *Collema subnigrescens* | NT | 8 | 2 | - | - |
|  | *Conotrema populorum* | DD | 3 | 2 | 1 | 1 |
|  | *Micarea globolusella*^d^ | NT | -^d^ | 19 | -^d^ | 14 |
|  | *Ramalina sinensis* | NT | 1 | 1 | - | - |
|  | *Rinodina degeliana* | VU | 3 | 3 | 1 | 1 |
| No. bryophyte observations |  |  | 188 |  | 38 |  |
| No. lichen observations |  |  | 360 |  | 438 |  |
| No. lichen observations excl. *B. nad* |  |  | 125 |  | 43 |  |

^a^ Gärdenfors (2000). DD = data deficient, EN = endangered, NT = near threatened, VU = vulnerable

^b^ One observation = presence in a 10 x 10 m square

^c^ Number of stands in which the species was found

^d^ This species was too common to be surveyed in detail in every stand. Only presence or absence of the species in the 100x100 m plot was record.

**References**

Gärdenfors, U., Ed. (2000). Rödlistade arter i Sverige 2000 (The 2000 Red List of Swedish species). Uppsala, ArtDatabanken, SLU.
